# In this Issue

**Published:** 2011

## FETAL ALCOHOL SPECTRUM DISORDERS: RESEARCH CHALLENGES AND OPPORTUNITIES

Although the adverse effects of prenatal alcohol exposure, which can result in a range of deficits collectively labeled fetal alcohol spectrum disorders (FASD), have long been known, challenges still remain in the identification and treatment of individuals with FASD. According to Dr. Kenneth R. Warren, Ms. Brenda G. Hewitt, and Dr. Jennifer D. Thomas, an important aspect of current FASD research is the development of tools to better identify individuals affected by prenatal alcohol exposure. Other efforts must focus on further elucidating the consequences of prenatal alcohol exposure, particularly neurodevelopmental deficits, and the mechanisms underlying alcohol’s detrimental effects. Understanding these mechanisms, as well as genetic and socioeconomic factors contributing to the risk for FASD, will allow researchers and clinicians to develop more targeted prevention approaches as well as more effective treatment strategies. (pp. 4–14)

## MATERNAL RISK FACTORS FOR FETAL ALCOHOL SPECTRUM DISORDERS: NOT AS SIMPLE AS IT MIGHT SEEM

Information about maternal drinking during pregnancy is vital for understanding the link between specific risk factors and a diagnosis along the continuum of fetal alcohol spectrum disorders (FASD); that information also can be the most difficult to obtain. Drs. Philip A. May and J. Phillip Gossage examine the importance of studying the drinking behaviors of women with children who have FASD. Risk factors such as the quantity, frequency, and timing of alcohol exposure are important but so are other factors, such as the mother’s age; the number of prior pregnancies and number of children she has had; her body size, nutrition, and metabolism; as well as her socioeconomic status, spirituality, mental health, use of other drugs, and social relationships. (pp. 15–26)

## COMBINATION DRUG USE AND RISK FOR FETAL HARM

It is well known that using alcohol, tobacco, and illicit drugs during pregnancy individually are hazardous to the developing fetus. When these are used in combination, however, the effects can be even more challenging and unpredictable. This short article by Drs. Wei-Jung A. Chen and Susan E. Maier describes the way drugs are absorbed, distributed, metabolized, and eliminated (i.e., pharmacokinetics) and how one drug can seriously and unpredictably alter the concentration, bioavailability (the rate of a drug entering the bloodstream), and net effect of the other drug. (pp. 27–28)

## EPIGENETICS AND FETAL ALCOHOL SPECTRUM DISORDERS

Epigenetics refers to stable but potentially reversible alterations in a cell’s genetic information that result in changes in gene expression but do not involve changes in the underlying DNA sequence. Animal studies now suggest that epigenetic changes resulting from a mother’s alcohol use may affect various stages of development and contribute to some of the abnormalities associated with fetal alcohol spectrum disorders. Drs. Michael S. Kobor and Joanne Weinberg look at the ways in which alcohol exposure may induce epigenetic changes prior to and during pregnancy, affecting critical pathways that affect gene expression. (pp. 29–37)

## UNDERSTANDING THE EFFECTS OF PRENATAL ALCOHOL EXPOSURE USING THREE-DIMENSIONAL FACIAL IMAGING

Although the facial features associated with fetal alcohol syndrome (FAS) can be distinct, some children with FAS may not be readily identified unless seen by a trained specialist. Moreover, subtle facial features may be present in people within the continuum of FASD. This brief article by Ms. Leah Wetherill and Dr. Tatiana Foroud examines a new three-dimensional imaging tool for detecting subtle facial characteristics associated with prenatal alcohol exposure. (pp. 38–41)

## DISCRIMINATING THE EFFECTS OF PRENATAL ALCOHOL EXPOSURE FROM OTHER BEHAVIORAL AND LEARNING DISORDERS

Because fetal alcohol spectrum disorders (FASD) is not a medical diagnosis but rather a description of a spectrum of the consequences of prenatal alcohol exposure, it may be under recognized, especially in general clinical settings where children are referred for diagnosis and treatment of developmental and behavioral problems. This article by Dr. Claire D. Coles reviews the neurodevelopmental effects of prenatal alcohol exposure, including alterations in learning and memory, motor and sensory/motor skills, visual/spatial skills, and executive functioning and self-control. Appropriate identification of the behavioral and developmental problems associated with FASD will allow for more targeted and effective interventions. (pp. 42–50)

## THE QUEST FOR A NEUROBEHAVIORAL PROFILE OF HEAVY PRENATAL ALCOHOL EXPOSURE

Drinking heavily during pregnancy can have devastating results for the growing fetus, ranging from full-blown fetal alcohol syndrome (FAS), with its recognizable physical characteristics, to a variety of subtle neurobehavioral effects. In this short article, Drs. Sarah N. Mattson and Edward P. Riley describe the importance of developing a profile that takes into account the range of neurobehavioral effects found with heavy prenatal alcohol exposure. Such a profile would improve the identification and, consequently, the treatment of people with fetal alcohol spectrum disorders, even those who do not show the typical physical characteristics of FAS. (pp. 51–55)

## BIOMARKERS OF FETAL ALCOHOL EXPOSURE AND FETAL ALCOHOL EFFECTS

Methods to screen for maternal drinking during pregnancy often can be inaccurate, and there are limitations of currently established biomarkers of alcohol consumption among pregnant women and alcohol exposure among newborns, making it difficult to accurately identify children with fetal alcohol spectrum disorders (FASD). Drs. Ludmila N. Bakhireva and Daniel D. Savage review the strengths and limitations of existing biomarkers and look at recent efforts to identify markers that can more accurately indicate the timing, duration, and level of prenatal alcohol exposure. (pp. 56–63)

## BEHAVIORAL INTERVENTIONS FOR CHILDREN AND ADOLESCENTS WITH FETAL ALCOHOL SPECTRUM DISORDERS

Drinking during pregnancy can lead to a constellation of effects, termed fetal alcohol spectrum disorders (FASD), that range from intellectual and learning disabilities, poor executive function, and speech and language delays, to behavioral and emotional difficulties, poor social skills, and motor deficits. Drs. Blair Paley and Mary J. O’Connor examine the usefulness and the challenges associated with behavioral interventions in people with FASD. Such approaches include parent-focused interventions, educational and cognitive interventions, and adaptive skills training. The article also reviews recent studies on the efficacy of behavioral approaches and highlights potentially fruitful directions for future research on the treatment of FASD. (pp. 64–75)

## FETAL ALCOHOL SPECTRUM DISORDERS: EXPERIMENTAL TREATMENTS AND STRATEGIES FOR INTERVENTION

Drinking during pregnancy is known to cause a broad range of physical, neurological, and behavioral alterations, collectively known as fetal alcohol spectrum disorders (FASD), yet many women continue to drink. This has given rise to the need for experimental models to identify potential treatments for FASD. This article by Drs. Nirelia M. Idrus and Jennifer D. Thomas reviews different experimental treatments—such as pharmacological, nutritional, and environmental/behavioral interventions—that can help mitigate some of the effects of prenatal alcohol exposure. Certain treatments target the underlying mechanisms that contribute to alcohol-induced damage, protecting against alcohol’s teratogenic effects, whereas others may act to enhance plasticity in the central nervous system, either during alcohol exposure or long after alcohol exposure has ceased. (pp. 76–85)

## PRENATAL ALCOHOL EXPOSURE AND MISCARRIAGE, STILLBIRTH, PRETERM DELIVERY, AND SUDDEN INFANT DEATH SYNDROME

Anumber of adverse pregnancy and birth outcomes have been linked to prenatal alcohol exposure. Studies suggest that drinking during pregnancy may increase the risk of miscarriage, stillbirth, preterm delivery, and sudden infant death syndrome. Drs. Beth A. Bailey and Robert J. Sokol review this research as well as the limitations of these studies. Establishing how combinations of alcohol’s biological actions, as well as sociodemographic and other comorbid conditions, contribute to these adverse outcomes is an ongoing challenge. (pp. 86–91)

## THE USE OF ANIMAL MODELS FOR THE STUDY OF FETAL ALCOHOL SPECTRUM DISORDERS

Studies in animals confirmed the relationship between prenatal alcohol exposure and neurodevelopmental disorders, which were first identified in human observational studies. Drs. Shannon E. Wilson and Timothy A. Cudd review the use of animal models in three areas of research: addressing basic questions about alcohol exposure during development; improving the identification of affected individuals; and developing approaches to reduce the impact of prenatal alcohol exposure. These animal model systems each provide specific strengths and together have helped to advance the research field of fetal alcohol spectrum disorders. (pp. 92–98)

## MAGNETIC RESONANCE–BASED STUDIES OF FETAL ALCOHOL SPECTRUM DISORDERS IN ANIMAL MODELS

Magnetic resonance–based imaging technologies, including magnetic resonance imaging, diffusion tensor imaging, and magnetic resonance spectroscopy, are useful tools for studying the effects of alcohol on the developing brain and have important implications for clinical practice and research. Drs. Shonagh K. O’Leary-Moore, Scott E. Parnell, Elizabeth A. Godin, and Kathleen K. Sulik show how these technologies are revealing the consequences of prenatal alcohol exposure in rodents, where alcohol exposure parameters, such as dose, timing and duration of alcohol, can be manipulated. The authors note that alcohol can induce neuropathology even at very early developmental stages, prior to the time when pregnancy typically is recognized in humans. Relating alcohol exposure parameters with developmental outcomes can improve diagnosis and prevention of FASD. (pp. 99–105)

## NEUROTRANSMITTER SYSTEMS

Prenatal alcohol exposure has been linked to abnormalities in the formation and refinement of developing brain circuits that are likely to be, in part, responsible for the persistent brain dysfunction and behavioral effects that characterize fetal alcohol spectrum disorders (FASD). Dr. C. Fernando Valenzuela, Mr. Michael P. Puglia, and Mr. Stefano Zucca describe recent research using animal models of FASD to examine the effects of developmental ethanol exposure on γ-aminobutyric acid (GABA), glutamate, serotonin, and dopamine systems. The studies reviewed involve diverse animal models and examine both short- and long-term effects on brain chemistry and function. (pp. 106–120)

## STRUCTURAL AND FUNCTIONAL BRAIN ABNORMALITIES IN FETAL ALCOHOL SPECTRUM DISORDERS

Brain imaging studies of children and adolescents with fetal alcohol spectrum disorders (FASD) have detected differences in brain structure related to alcohol exposure in multiple brain systems and abnormalities in the white matter that connects these brain regions. Dr. S. Christopher Nuñez, Ms. Florence Roussotte, and Dr. Elizabeth Sowell review research on the relationships between these morphological differences and important cognitive functions, such as working memory, learning, and inhibitory control. (pp. 121–131)

